# Neuromelanin-induced cellular stress and neurotoxicity in the pathogenesis of Parkinson's disease

**DOI:** 10.1007/s10495-025-02156-3

**Published:** 2025-08-07

**Authors:** Md. Jakaria, Jason R. Cannon

**Affiliations:** 1 School of Health Sciences, Purdue University, West Lafayette, IN 47907 USA; 2 Purdue Institute for Integrative Neuroscience, Purdue University, West Lafayette, IN 47907 USA

**Keywords:** Neuromelanin, Interaction, Toxic compounds, Cellular stress and neurodegeneration

## Abstract

Neuromelanin is a complex dark brown pigment that primarily accumulates in catecholaminergic neurons, particularly in the substantia nigra and locus coeruleus regions of the brain in primates. Rats and mice are largely devoid of neuromelanin, although it is present in some other non-primate species. This pigment is notable for its age-related accumulation and has been linked to the pathophysiology of various neurodegenerative diseases, especially Parkinson's disease. Research has increasingly suggested that neuromelanin or its precursors trigger cellular stress, including neuroinflammation, apoptosis, oxidative stress, mitochondrial dysfunction, and impaired autophagy. Collectively, these mechanisms significantly contribute to neurodegeneration. Additionally, neuromelanin can interact with various neurotoxic molecules, potentially forming complexes that may provide protective benefits against neurotoxicity. However, extensive studies also suggest that this interaction can have a double-edged effect; while it may sequester harmful substances, it can simultaneously increase cellular stress and enhance neuronal toxicity, creating a detrimental cycle. We review the multifaceted roles of neuromelanin in the brain, discussing how its properties and interactions contribute to cellular stress and the progression of neurodegenerative processes. In the context of neurotoxic mechanisms, we also address potential therapeutic targets for Parkinson’s disease.

## Introduction

**Fig. 1:**
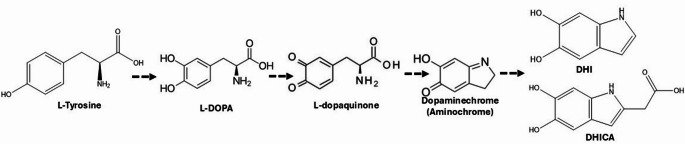
Essential catecholamine building blocks of eumelanin/neuromelanin formation

Neuromelanin is a type of melanin that is a dark brown organic pigment that forms over time from the metabolites of catecholamine neurotransmitters and consists of a mixture of eumelanin and pheomelanin, including covalently bound lipids, peptides, and metals [1, 2]. Eumelanin consists of 5,6-dihydroxyindole (DHI) and 5,6-dihydroxyindole-2-carboxylic acid (DHICA) moieties, which can be formed from a series of conversions initiated from L-tyrosine (Fig. 1), whereas pheomelanin consists of benzothiazine (BT) and benzothiazole (BZ) moieties. In vivo neuromelanin formation is still not completely understood. Tyrosinase is ectopically used to form neuromelanin in model systems, and it may have a role in human formation, although its presence in the human brain remains controversial [3–5]. Other enzymes, such as prostaglandin H synthase, peroxidase, and macrophage migration-inhibitory factors, are also involved in neuromelanin synthesis [5–7]. Additionally, neuromelanin can form through non-enzymatic oxidation, including the autoxidation of catecholamines (catechols oxidizes to quinones followed by thiol group addition) in the brain [5, 8].

Neuromelanin is thought to have dual roles with respect to toxicity: it may provide neuroprotection by binding to toxic substances, but it can also be neurotoxic. A recent study found that neuromelanin-like nanoparticles can protect neurons by scavenging reactive oxygen species (ROS). However, these nanoparticles can also lead to neuronal cell death due to excessive binding to iron ions [9]. Neuromelanin was also shown to bind to glutathione peroxidase 4 (GPX4, a selenoprotein that principally detoxifies lethal phospholipid hydroperoxides) in the human substantia nigra [10]. This binding suggests a neuromelanin role in cellular stress.

Parkinson's disease (PD) is the second most common neurodegenerative disease and a major cause of mortality and morbidity worldwide. The pathogenesis of PD primarily involves with the progressive degeneration and death of dopaminergic neurons in the substantia nigra pars compacta (SNpc) region of the brain, though other brain regions, cell types and bodily systems are affected. Another major pathological hallmark of PD is the presence of Lewy bodies, which are protein aggregates primarily composed of α-synuclein [11]. The loss of dopaminergic neurons leads to a significant decrease in dopamine levels. The neurotransmitter dopamine is crucial for motor control; as a result, PD causes an imbalance in brain activity, especially within the basal ganglia, contributing to characteristic motor symptoms such as tremors, rigidity, and slow movement [11]. Several risk factors for PD have been identified, including aging, genetics, and environmental exposure to toxic substances [12, 13]. Neuroinflammation, impaired autophagy, and multiple cellular stressors such as mitochondrial stress, apoptosis, and oxidative stress are implicated in the pathogenesis of PD [14, 15]; however, the precise cause of neurodegeneration in this condition remains unknown. Currently, there is no cure for PD, and available treatments focus on alleviating symptoms.

Neuromelanin has been implicated in the pathogenesis of PD. Importantly, as we age, the accumulation of neuromelanin affects the pathogenesis of PD. Neurons that contain neuromelanin, particularly the dopaminergic subpopulations with the highest levels, are especially vulnerable to neurodegeneration [16, 17]. Evidence also suggests that neuromelanin accumulation can lead to intracellular aggregation of endogenous α-synuclein, defects in autophagy and ubiquitin-proteasome system (UPS) [18–20], which can cause neurodegeneration. In addition to intracellular neuromelanin, secreted neuromelanin from dying neurons can trigger neurotoxicity. Exogenous neuromelanin can trigger neuroinflammation as a response to cellular stress, contributing to neurodegeneration in models of PD [21, 22]. Furthermore, neuromelanin can induce various forms of cellular stress, including mitochondrial dysfunction, oxidative stress, and apoptosis, in neuronal cultures [23, 24], which may contribute to the underlying mechanisms of neurodegeneration. Neuromelanin can also bind to neurotoxins and neurotoxicants, such as L-methyl-4-phenylpyridine (MPP^+^), 2-amino-1-methyl-6-phenylimidazo[4,5-b]pyridine (PhIP), and harmane [25–27]. However, the complex interactions between neuromelanin and these toxic compounds, which contribute to cellular stress and neurotoxicity, are not fully understood. This review discusses the mechanisms through which neuromelanin induces cellular stress and toxicity, thereby exerting a significant influence on the progression of neurodegenerative diseases, particularly PD.

## Contribution of neuromelanin to cellular stress and neurodegeneration

### Impacts of dopamine oxidation and neuromelanin precursor aminochrome

**Fig. 2:**
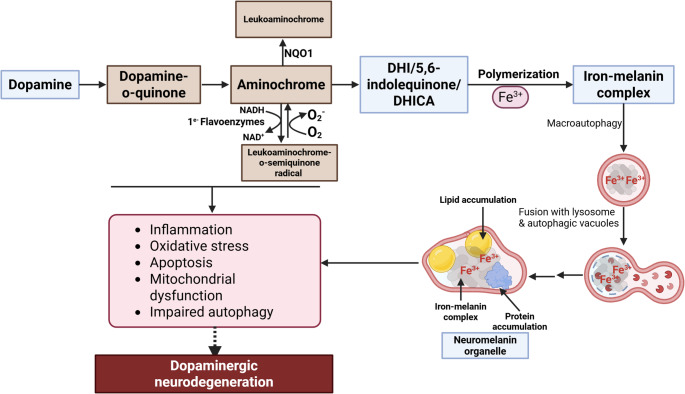
The proposed mechanism by which neuromelanin mediates neurotoxicity in dopaminergic neurons. Free dopamine can oxidize to form aminochrome [33]. NQO1 (DT-diaphorase) catalyzes the two-electron reduction of aminochrome to leukoaminochrome, which prevents the neurotoxic effects associated with aminochrome. The presence of dioxygen can lead to slow oxidation of leukoaminochrome, generating superoxide. This autoxidation occurs much more rapidly when superoxide is present. When aminochrome is reduced by a single electron, it results in a redox cyclization between aminochrome and the leukoaminochrome o-semiquinone radical, which depletes dioxygen and NADH (nicotinamide adenine dinucleotide (NAD) + hydrogen (H)). This depletion of NADH impacts the activity of the mitochondrial respiratory chain, ultimately affecting ATP production [33]. Aminochrome can further oxidize to generate DHI, 5,6-indolequinone, and DHICA. This process initiates oxidative polymerization, creating a melanin-protein component comprising both eumelanin and pheomelanin moieties, which can bind large amounts of metals, particularly iron. Through macroautophagy, the resulting undegradable material is transported into autophagic vacuoles, where it fuses with lysosomes and other autophagic vacuoles containing lipid and protein components. This fusion ultimately forms organelles that contain neuromelanin, various metals, abundant lipid bodies, and a protein matrix [34]. Neuromelanin and its precursors, dopamine o-quinone and aminochrome, can induce cellular stress, leading to neurotoxicity and neurodegeneration in dopaminergic neurons

Dopamine is initially catabolized through oxidation (intracellular) or methyl transferase (extracellular) activity, both pathways eventually converge to form homovanillic acid as the final metabolite [28]. Alternate pathways produce neuromelanin, where dopamine can be oxidized through two main processes: tyrosinase activity or autoxidation (Fig. 2). This oxidation results in the formation of dopamine o-quinone, an unstable intermediate. Dopamine oxidation has been linked to cellular stress, such as oxidative stress, lipid peroxidation, apoptosis, and mitochondrial and lysosomal dysfunction (Fig. 2), which are factors that contribute to the pathogenesis of PD [29–32].

Under physiological pH conditions, dopamine o-quinone rapidly converts to aminochrome, a more stable cyclic compound. This transformation is a subsequent step in the oxidation pathway of dopamine; thus, dopamine o-quinone quickly transitions to aminochrome within the body. Subsequently, aminochrome is rearranged to DHI, which is then oxidized to 5,6-indolequinone, which ultimately polymerizes to form neuromelanin (Fig. 2) [34, 35].

Aminochrome can be converted into leukoaminochrome-o-semiquinone radical, which is a one-electron reduced form of aminochrome, through the action of flavoenzymes. This radical is highly reactive, suggesting that the toxicity of aminochrome may be linked to its radical formation [36].

DT-diaphorase (NQO1) is a flavoprotein found in dopaminergic neurons and astrocytes. NQO1 is capable of reducing aminochrome and is the only flavoenzyme known to catalyze the two-electron reduction of quinones [37]. Research has indicated that NQO1 can prevent the one-electron reduction of aminochrome to leukoaminochrome-o-semiquinone radical by facilitating its two-electron reduction to leukoaminochrome [38, 39].

Aminochrome has been shown to induce various forms of cellular stress, leading to neurotoxicity in dopaminergic neurons (Fig. 2). It has been found to cause inflammation in organotypic midbrain slice cultures and rat dopaminergic neurons [40, 41]. Additionally, it decreases the neurotrophic factors NGF (nerve growth factor) and GDNF (glial-derived neurotrophic factor) in these midbrain slice cultures [40]. Aminochrome binds to α-synuclein, enhancing the formation and stabilization of its protofibrils [42, 43]. It has also been found to increase toxicity caused by α-synuclein oligomers in RCSN-3 cells (a catecholaminergic cell line derived from the substantia nigra of an adult rat) [43] and cause toxicity by impairing autophagy and lysosomal function in glioblastoma cells [44]. Furthermore, it leads to mitochondrial dysfunction, as indicated by the inhibition of mitochondrial complex I and a reduction in ATP production in differentiated SH-SY5Y neuroblastoma cells (a dopaminergic phenotype) [45]. This dysfunction leads to a consistent decrease in ATP levels in the brains of rats. These data also explain the significant reduction in dopamine release and the number of synaptic vesicles at the synaptic cleft, as the transport of neurotransmitter vesicles to the terminals and the release of dopamine are heavily dependent on ATP [46].

Moreover, aminochrome is involved in oxidative stress; it increases the expression of the iron import transporter, divalent metal transporter 1, while decreasing the expression of the iron export transporter, ferroportin 1 [45]. This finding is also consistent with an increase in iron uptake with aminochrome treatment [45], thereby aminochrome may contribute to oxidative stress. Additionally, aminochrome induces apoptosis, and the activation of autophagy can mitigate this effect [47].

### Effects of tyrosinase overexpression: an enzyme involved in the neuromelanin biosynthetic pathway

The overexpression of human tyrosinase leads to the accumulation of neuromelanin, resulting in cellular stress and neurodegeneration in pre-clinical studies. Tyrosinase overexpression increases intracellular dopamine and melanin formation in neuronal somata, resulting in apoptosis in SH-SY5Y cells [48]. Recent studies have shown that human tyrosinase overexpression causes neurodegeneration in rodents [18, 49–51]. Human tyrosinase overexpression in rat substantia nigra leads to age-related production of human-like neuromelanin in dopaminergic neurons, reaching levels seen in elderly humans. This accumulation is associated with an age-dependent PD phenotype, marked by hypokinesia and neurodegeneration in the nigrostriatal pathway [18]. However, tyrosinase overexpression causes neuromelanin formation in pre-clinical studies, but the expression of tyrosinase in the brain is very low [18, 52, 53]. Therefore, the ectopic tyrosinase expression may reliably produce human-relevant neuromelanin, though it may do so through biochemical pathways different from those driving neuromelanin formation in the human brain.

### Direct impacts of neuromelanin

Beyond the impact of neuromelanin formation, exogenous neuromelanin has been shown to contribute to cellular stress, including oxidative stress, mitochondrial dysfunction, and apoptosis (Fig. 2) [18, 21, 23, 24, 54], which can cause dopaminergic neurodegeneration. Research has shown that neuromelanin increases oxidative stress, as marked by elevated levels of reactive oxygen and nitrogen species in isolated mitochondria from SH-SY5Y cells. This oxidative stress was ameliorated through the treatment with the iron chelator deferoxamine and the antioxidants superoxide dismutase and (-)-epigallocatechin gallate [23]. Neuromelanin has also been shown to impair mitochondria and induce apoptosis in SH-SY5Y cells, as evidenced by a decrease in mitochondrial membrane potential, an increase in cytochrome c release, and caspase-3 activation. This study highlights how neuromelanin reduces the levels of S-glutathionylated proteins in the mitochondria and disrupts the macromolecular structure of complex I. The involvement of reactive oxygen and nitrogen species is crucial for the deglutathionylation process induced by neuromelanin. Additionally, antioxidants significantly mitigate this effect and help prevent apoptosis [24].

Growing evidence indicates that neuromelanin also activates specific immune cells, including microglia, astrocytes, and dendritic cells [22, 55–58]. Research has shown that neuromelanin can trigger inflammation through the NF-kappa B-dependent and p38 mitogen-activated protein kinase-dependent pathways in primary microglia derived from rats [56]. Additionally, delivering neuromelanin directly into the brain (via intracerebral injection) has been found to induce inflammation in the substantia nigra of rats [22]. The impact of neuromelanin on neuroinflammation was associated with neurotoxicity. Neuromelanin caused greater neurotoxicity, indicated by decreased dopamine uptake, when microglia were present, compared to neuron-enriched cultures [57]. Neuromelanin also activates caspase-8-dependent cellular stress, including microglial activation and oxidative stress, in BV-2 microglia. Treatment with a caspase-8 inhibitor attenuated this cellular stress [21].

In addition to neuroinflammation, neuromelanin has been shown to hinder both proteasomal activity and autophagic clearance. The accumulation of neuromelanin within autophagic compartments in SH-SY5Y cells that overexpress tyrosinase is linked to a decrease in lysosomal-mediated proteolysis and a significant reduction in the activity of the UPS [18, 19]. Specifically, neuromelanin impairs the UPS by inhibiting the enzymatic activity of the 26S proteasome [19]. Studies involving human subjects have revealed that both autophagy and the UPS are impaired in regions of post-mortem PD brains that contain neuromelanin compared to non-melanized areas [59–61].

## Impact of the interaction between neuromelanin and toxic/pathogenic molecules

**Fig. 3:**
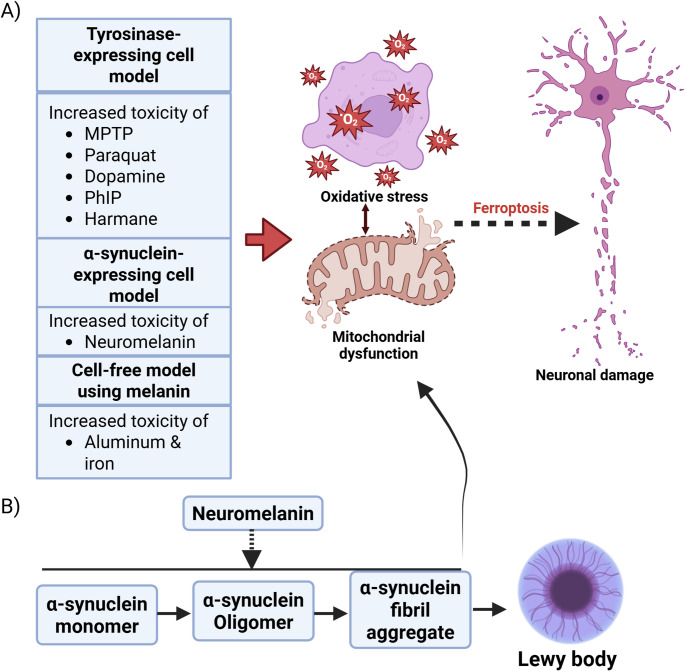
Common toxic compound-induced cellular stress in experimental models. The interaction between neuromelanin and toxicants can increase cellular stress, including oxidative stress and mitochondrial dysfunction. These factors may result in ferroptotic cell death that contributes to damage in dopaminergic neurons (**A**). Neuromelanin can increase the aggregation of α-synuclein fibrils, potentially increasing cellular stress and toxicity (**B**)

Data from multiple species and experimental approaches show that neuromelanin has a significant impact on neurotoxicant uptake, where cells that form neuromelanin have higher toxicant levels. Specifically, neuromelanin can modulate internal concentration, whereby neuromelanin-positive catecholaminergic neurons can have higher internal concentrations of toxicants. Experiments on several toxicants suggest that such interactions occur across broad chemical classes and that binding is not protective, but rather, increases toxicity in pre-clinical models. The relationship between neuromelanin and toxic molecules can contribute to stress and toxicity in dopaminergic neurons (Fig. 3).

### Alpha-synuclein

Multiple studies have revealed interactions between α-synuclein and neuromelanin in the context of PD pathogenesis. In healthy tissue, neuromelanin granules contain α-synuclein [20, 62–64] and neuromelanin has been shown to increase the expression, accumulation, or aggregation of α-synuclein in the dopaminergic neurons of the substantia nigra in aging or PD brains [20, 62–64]. In PD, α-synuclein has been shown to translocate into lipid droplets in nigral neurons, but this does not occur in the ventral tegmental area [64]. Furthermore, mutant forms of α-synuclein associated with PD have been specifically localized to lipid droplets, leading to a loss of neutral lipid staining [65]. However, further studies are needed to determine whether the influence of α-synuclein on the lipid levels of neuromelanin affects the stress and neurotoxicity of dopaminergic neurons.

Additionally, α-synuclein overexpression has been demonstrated to enhance melanin content in SH-SY5Y and PC12 cells, suggesting that α-synuclein may promote neuromelanin synthesis [66]. In a recent study, human α-synuclein overexpression in the mouse substantia nigra was found to elevate striatal dopamine levels [67]. Thus, increased α-synuclein levels may enhance neuromelanin synthesis by increasing dopamine levels. It is also possible that neuromelanin-induced oxidative stress triggers α-synuclein accumulation, or neuromelanin accumulation might lead to impaired autophagy, resulting in reduced α-synuclein clearance. The exact mechanism by which neuromelanin influences, or is influenced by, α-synuclein levels remains unclear.

Growing evidence suggests that α-synuclein has an impact on cellular stress and neurotoxicity. Misfolded α-synuclein can trigger apoptosis by acting on caspase-3 signaling [68]. Oligomeric and fibrillar α-synuclein have been shown to induce oxidative stress by increasing ROS production and disrupting phospholipid membranes [68, 69]. Ferroptosis is a type of non-apoptotic and uncontrolled cell death characterized by oxidative stress and impaired phospholipid peroxidation [70, 71]. This cell death has been implicated as a mechanism of neurodegeneration [72]. Research has shown that α-synuclein aggregation induces ferroptosis, and α-synuclein expression increases susceptibility to ferroptosis in neurons [73, 74]. The pentose phosphate pathway plays a crucial role in managing oxidative stress by generating NADPH, a reducing agent that helps cells combat harmful ROS. However, the accumulation of α-synuclein microaggregates has been found to impair this important pathway [69].

Moreover, α-synuclein can contribute to neuroinflammation-associated neurotoxic damage. Research has shown that α-synuclein fibrils from PD patients, in conjunction with tumor necrosis factor alpha and prostaglandin E2, stimulate microglial cell activation. This leads to a specific and highly neurotoxic chronic inflammatory phenotype characterized by significant glutamate release and iron retention [75]. Therefore, the impact of neuromelanin on α-synuclein expression, accumulation or aggregation can impact cellular stress and neurotoxicity. Indeed, the number of dopaminergic neurons decreases with aging, which is linked to the accumulation of neuromelanin and α-synuclein [62]. The impact of neuromelanin on α-synuclein-associated toxicity has also been observed in SK-N-SH cells, a human neuroblastoma cell line [54]. Neuromelanin significantly enhances the toxicity of the Fenton reaction, which consists of 400 μM FeSO_4_ and 200 μM H2O2, in SK-N-SH cells that express α-synuclein, whether wild-type or carry the A53T mutation. This toxicity is linked to cellular stress, as indicated by decreased cell viability, increased apoptosis, and heightened oxidative stress, demonstrated by elevated hydroxyl radical production and lipid peroxidation [54]. These evidences recommend further investigation into whether and how neuromelanin and α-synuclein directly interact to drive neurotoxicity and neurodegeneration in PD.

### MPP+

Methylphenylpyridine (MPP+) is a neurotoxicant and a metabolic byproduct of MPTP (1-methyl-4-phenyl-1,2,3,6-tetrahydropyridine), formed through the action of the enzyme monoamine oxidase B. MPTP is not generally considered a common environmental risk; rather, it is primarily recognized as a contaminant found in certain drugs. The Parkinsonism-like symptoms caused by MPTP were first identified following an accidental injection related to contaminated 1-methyl-4-phenyl-4-propionoxy-piperidine (MPPP) [76, 77]. MPPP is a synthetic opioid drug that has effects comparable to those of morphine and pethidine (meperidine). MPP+ selectively accumulates in dopamine neurons via the dopamine transporter, leading to its accumulation and subsequent dopaminergic neurodegeneration by causing mitochondrial dysfunction via inhibiting mitochondrial complex I. Both MPTP and MPP+ have proven to be valuable tools in studying dopaminergic neurodegeneration in PD.

MPP+ is known to bind with high affinity to both melanin and neuromelanin [25]. Since MPP+ binds with neuromelanin, MPP+ could gradually release to cause progressive damage to neurons [25]. It is also possible that neuromelanin-MPP+ bound neurons can further be susceptible to damage by additional toxicants. Indeed, an early study demonstrated that neuromelanin-containing dopaminergic neurons in cynomolgus monkeys are more susceptible to MPTP toxicity than non-melanized neurons [78]. In addition to MPTP/MPP+, the effects of interactions between neuromelanin and other PD toxicants such as 6-hydroxydopamine can be studied since an early study demonstrated that tyrosinase overexpression increases the sensitivity to toxicity caused by dopamine and hydrogen peroxide in M17 cells, a neuroblastoma cell line [53].

### Paraquat

Paraquat is an herbicide that has a structure similar to MPP+. It can cause selective toxicity in dopaminergic neurons via the dopamine transporter, ultimately leading to mitochondrial dysfunction and oxidative stress. Paraquat has been shown to accumulate in neuromelanin and demonstrate an affinity for it [79, 80]. After an intraperitoneal injection of [14C]paraquat, accumulation and retention of neuromelanin were observed in frogs [79]. However, this affinity is considerably lower than that of MPP+ and certain antimalarial drugs when assessed in isolated natural neuromelanin [80]. While paraquat has been shown to increase cytotoxicity in tyrosinase-overexpressing M17 cells [53], further study is required to understand whether the interaction between environmentally relevant exposure to paraquat and neuromelanin worsens dopaminergic neurodegeneration.

### Heterocyclic aromatic amines

Heterocyclic aromatic amines (HAAs) are chemical compounds typically formed when protein-rich foods, such as meat and fish, are cooked at high temperatures. These compounds can also be found in other sources, such as tobacco smoke, heated cooking oils, and diesel exhaust [81]. HAAs have been extensively studied in cancer research due to their mutagenic properties, and they also have neurotoxic effects [82, 83]. HAAs such as harmane and PhIP were shown to bind to neuromelanin analogs Sepia melanin and dopamine melanin [27]. It was found that a maximum of 217 µg and 622 µg can bind to Sepia melanin and dopamine melanin, respectively. In contrast, a maximum of 65 µg and 189 µg of PhIP can bind to 1 mg of Sepia melanin and dopamine melanin, respectively [27].

In SH-SY5Y cells, which have been genetically modified to express human tyrosinase and are capable of forming neuromelanin, increased toxicity has been observed following treatment with HAA compounds such as harmane and PhIP [27, 84]. These compounds have been shown to increase cellular stress, as evidenced by heightened cytotoxicity, mitochondrial dysfunction, and oxidative stress [27, 84]. While these dietary toxicants demonstrated toxicity in cell culture models, further studies are needed in animal models to understand the mechanisms and extent of toxicity from dietary toxicants and neuromelanin interactions.

### L-BMAA

The cyanobacterial amino acid beta-N-methylamino-L-alanine (L-BMAA) is a neurotoxin linked to the development of neurodegenerative diseases. The primary mechanism underlying the neurotoxicity of L-BMAA is associated with increased excitotoxicity and oxidative stress [85]. Research has demonstrated that L-BMAA interacts with neuromelanin; specifically, a tritiated form of L-BMAA (3H-BMAA) is retained in neurons containing neuromelanin in frogs and binds to Sepia melanin. Additionally, the interaction of 3H-BMAA with melanin is stronger during melanin synthesis than when 3H-BMAA is bound to preformed melanin [86].

### Metals

Neuromelanin has been shown to interact with several heavy metals, including iron, copper, aluminum, lead, mercury, chromium, molybdenum, cadmium, and zinc [87, 88]. These heavy metals are known for their environmental toxicity.

The interaction between neuromelanin and metals can increase cellular stress and dopaminergic neurotoxicity. Neuromelanin has a high affinity for iron and may serve as a source of iron in the brain. Under conditions of iron overload, such as in PD, the binding sites on neuromelanin may become saturated, resulting in an increased presence of redox-active iron in the neuromelanin of the substantia nigra in PD patients [89, 90]. While iron is essential for brain function, dysregulation in iron levels can lead to oxidative stress and ferroptotic cell death. Indeed, research has shown that iron and aluminum can enhance melanin-induced lipid peroxidation [91]. This evidence suggests that interactions between metals and neuromelanin can lead to cellular stress such as ferroptosis and neurotoxicity. However, further study is needed to determine whether metals other than iron and aluminum bind to neuromelanin, potentially causing dopaminergic neurotoxicity. It is also necessary to investigate the conditions under which metal-neuromelanin interactions may be protective or harmful to cells.

## Targeting neuromelanin and neuromelanin-toxic compound interactions for neuroprotective therapy

Given the role of neuromelanin in binding with toxic compounds, as well as its involvement in promoting cellular stress and neurodegeneration, controlling neuromelanin levels and reducing the impairment caused by neuromelanin or its interactions with toxic compounds could offer a potential neuroprotective therapy for PD. Importantly, neuroprotective therapies could prevent the damaging effects of neuromelanin released from dying neurons, as this released neuromelanin can contribute to neurodegeneration. The following approaches have been discussed for reducing neuromelanin levels and the associated impairments:

### Ferroptosis inhibitors

Dopamine metabolism and neuromelanin play critical roles in cellular stress, particularly oxidative stress, which can lead to cell death through ferroptosis. Therefore, preventing oxidative stress-mediated ferroptosis could be a potential therapeutic approach for treating neurodegeneration associated with neuromelanin.

Since neuromelanin forms a complex with ferric iron, iron chelators, which act as ferroptosis inhibitors by chelating iron, can potentially be a therapeutic approach for dopamine metabolism and neuromelanin-associated neurodegeneration. Deferoxamine, an iron chelator, inhibits neuromelanin synthesis [92] and protects against dopamine-induced death in catecholaminergic cells [32]. An early study showed that deferasirox, another iron chelator and ferroptosis inhibitor, ameliorated neuromelanin-induced oxidative stress in isolated mitochondria [23].

In addition to preclinical studies, an iron chelator, deferiprone, has been shown to improve motor performance in a phase II clinical trial of PD [93]. However, in a subsequent study involving a large cohort of 372 participants—equally divided into placebo and deferiprone treatment groups—deferiprone did not demonstrate benefits for patients with early-stage PD [94]. In fact, this study indicated that deferiprone worsened Parkinsonism symptoms and was associated with adverse events. These negative effects may be attributed to deferiprone's inhibition of tyrosine hydroxylase, the rate-limiting enzyme in dopamine production, which aligns with the observed elevation of plasma prolactin, a substance that inhibits dopamine. Interestingly, despite these adverse effects, deferiprone treatment was found to preserve brain tissue in this trial [94]. This highlights the need for including dopamine therapy with deferiprone to reduce the negative effects of deferiprone.

While iron chelators show promise for PD therapy, further pre-clinical studies are necessary to understand whether iron chelators can effectively treat neuromelanin-associated dopaminergic neurodegeneration in PD.

Several ferroptosis inhibitors, including glutathione, N-acetyl-cysteine, dithiothreitol, retinol, vitamin E, and vitamin E analog Trolox, were found to be protective against dopamine-induced neuronal toxicity in early studies [32, 95], suggesting a possible neuroprotective role against neurodegeneration associated with dopamine metabolism, neuromelanin, and ferroptosis.

Testing tyrosinase inhibitors for neuromelanin-associated impairment may not be appropriate, as tyrosinase levels in the brain are very low. It is important to note that the neuroprotective effects of tyrosinase inhibitors, such as kojic acid, compounds 2-06 and S05014, might be linked to their anti-inflammatory and antioxidant properties [96–99].

Additionally, various antioxidant compounds, including retinoic acid, 5,6-dihydroxyflavone, and phenelzine, are recognized for their anti-ferroptotic properties [70, 71]. Indeed, these compounds have demonstrated neuroprotective effects against neuroinflammation, neuronal injury and neurodegeneration in pre-clinical studies [100–103]. Further research could test these compounds for their potential as neuroprotective therapies against dopaminergic cell loss.

### Inflammation and apoptosis inhibitors

Considering the role of neuroinflammation in neurodegeneration and the involvement of neuromelanin in this process, anti-inflammatory drugs hold potential as neuroprotective treatments for PD. A recent study found that dexamethasone, a glucocorticoid known for its strong anti-inflammatory properties, exhibits neuroprotective effects against damage related to neuromelanin formation in mice [104]. Dexamethasone not only significantly improved motor function but also helped preserve dopaminergic neurons. This preservation was linked to a reduction in reactive microglia and a decrease in the infiltration of peripheral immune cells into the brain [104].

Apoptosis is a significant indicator of damage associated with neuromelanin and plays a role in the neuromelanin-induced inflammatory response. Therefore, anti-apoptotic drugs could be explored alongside other neuroprotective therapies as potential treatments for neurodegeneration. Notably, an anti-apoptotic drug and caspase-8 inhibitor, IETD-fmk, has been shown to reverse inflammatory changes induced by neuromelanin. This finding suggests that anti-apoptotic drugs may be beneficial for addressing neuromelanin-related damage [21].

### Autophagy modulators

Given the pivotal detrimental role of impaired proteostasis in neuromelanin-laden cells [18], autophagy activation could be a strategy to prevent neuromelanin-associated toxicity. Indeed, enhancing lysosomal-mediated proteolysis by overexpressing transcription factor EB (TFEB) in neuromelanin-producing rats was shown to reduce the intracellular neuromelanin density, attenuate PD-like inclusion formation, prevent nigrostriatal neurodegeneration, and reverse motor impairment [18].

In addition, autophagy activation by rapamycin was shown to protect against aminochrome-, 6-OHDA and MPTP-induced neuronal cell death in PD models [47, 105]. Rapamycin, along with another autophagy inducer such as trehalose, a known activator of TFEB, was also shown to be protective against neurodegeneration in a PD model [106]. A clinical trial of rapamycin treatment for Alzheimer's disease (AD) is currently underway (https://clinicaltrials.gov/study/NCT04629495). This autophagy-inducing therapeutic approach shows promise for PD therapy by potentially decreasing the intracellular neuromelanin content, for instance, through its action on TFEB. This could lead to a reduction in the formation of α-synuclein aggregates and pathological inclusion bodies.

In addition to autophagy activators, autophagy inhibitors show promise in combating neurodegeneration related to neuromelanin. Chloroquine, an antimalarial drug that acts as an autophagy inhibitor, has been identified as the most effective competitor for MPP+ binding to melanin due to its high affinity for melanin. Furthermore, chloroquine has been found to protect against MPTP-induced dopaminergic neurodegeneration in monkeys [26]. Increasing evidence suggests that chloroquine also has neuroprotective effects against dopaminergic neurodegeneration and traumatic brain injury in cell culture and rodent models [107–109]. However, since chloroquine's neuroprotective effect is linked to the inhibition of autophagy, further research is needed to determine whether it exacerbates or benefits specific models involving neuromelanin formation.

### Modulation of miscellaneous targets for controlling neuromelanin-associated damage

FKBP51 (FK506-binding protein 51) is an immunophilin protein that functions as a peptidyl-prolyl isomerase (PPIase) and co-chaperone, playing key roles in protein folding, steroid receptor signaling, stress response, and metabolism [110]. FKBP51 is significantly upregulated with aging and in the brains of individuals with PD. Treatment with SAFit2, a potent FKBP51 inhibitor, was found to decrease ubiquitin-positive inclusions, prevent neurodegeneration in the substantia nigra, and improve motor function in NM-SNCAWT (neuromelanin-induced in humanized α-synuclein) mouse model [111].

DT-diaphorase has a protective function in astrocytes against aminochrome toxicity, since the inhibition of DT-diaphorase increases neuromelanin precursor aminochrome toxicity [43, 112, 113].

Vesicular monoamine transporter 2 (VMAT2) is a protein located in the membranes of synaptic vesicles within neurons. It plays a crucial role in the transport and storage of monoamine neurotransmitters, including dopamine, serotonin, norepinephrine, and histamine [114]. Defects in VMAT2-mediated dopamine encapsulation within synaptic vesicles have been observed in early PD patients [115]. Moreover, there is a negative correlation between neuromelanin levels and VMAT2 immunoreactivity in the dopaminergic neurons of the human midbrain. Neurons that exhibit the highest levels of VMAT2 and the lowest levels of neuromelanin are less prone to neurodegeneration associated with PD [116].

A recent study conducted with neuromelanin-producing rats found that overexpressing VMAT2 via a viral vector significantly improves the encapsulation of dopamine within vesicles in the substantia nigra [50]. This genetic approach reduces the formation of potentially harmful oxidized dopamine species, which can be converted into neuromelanin. As a result, it helps maintain intracellular neuromelanin levels below the pathogenic threshold. Furthermore, the decreased neuromelanin production in these rats was linked to a reduction in Lewy body-like inclusions and contributed to the long-term preservation of dopamine balance, the integrity of nigrostriatal neurons, and overall motor function [50].

In conjunction with the previously mentioned strategies, exploring active immunotherapy utilizing monoclonal antibodies presents a promising avenue for addressing the damage linked to neuromelanin. By harnessing the specificity and potency of these antibodies, this approach could mitigate the adverse effects associated with neuromelanin, paving the way for innovative treatment options.

## Conclusion

Research indicates that the formation and accumulation of neuromelanin are associated with dopaminergic neurodegeneration beyond its normal physiological roles. In PD, neuromelanin can be formed due to increased levels of free cytosolic dopamine, since this dopamine can oxidize into neuromelanin. However, the exact mechanisms underlying neuromelanin formation remain unclear. Further studies are needed to determine whether multiple enzymes and/or autoxidation of dopamine are required for neuromelanin production in the brain.

**Fig. 4:**
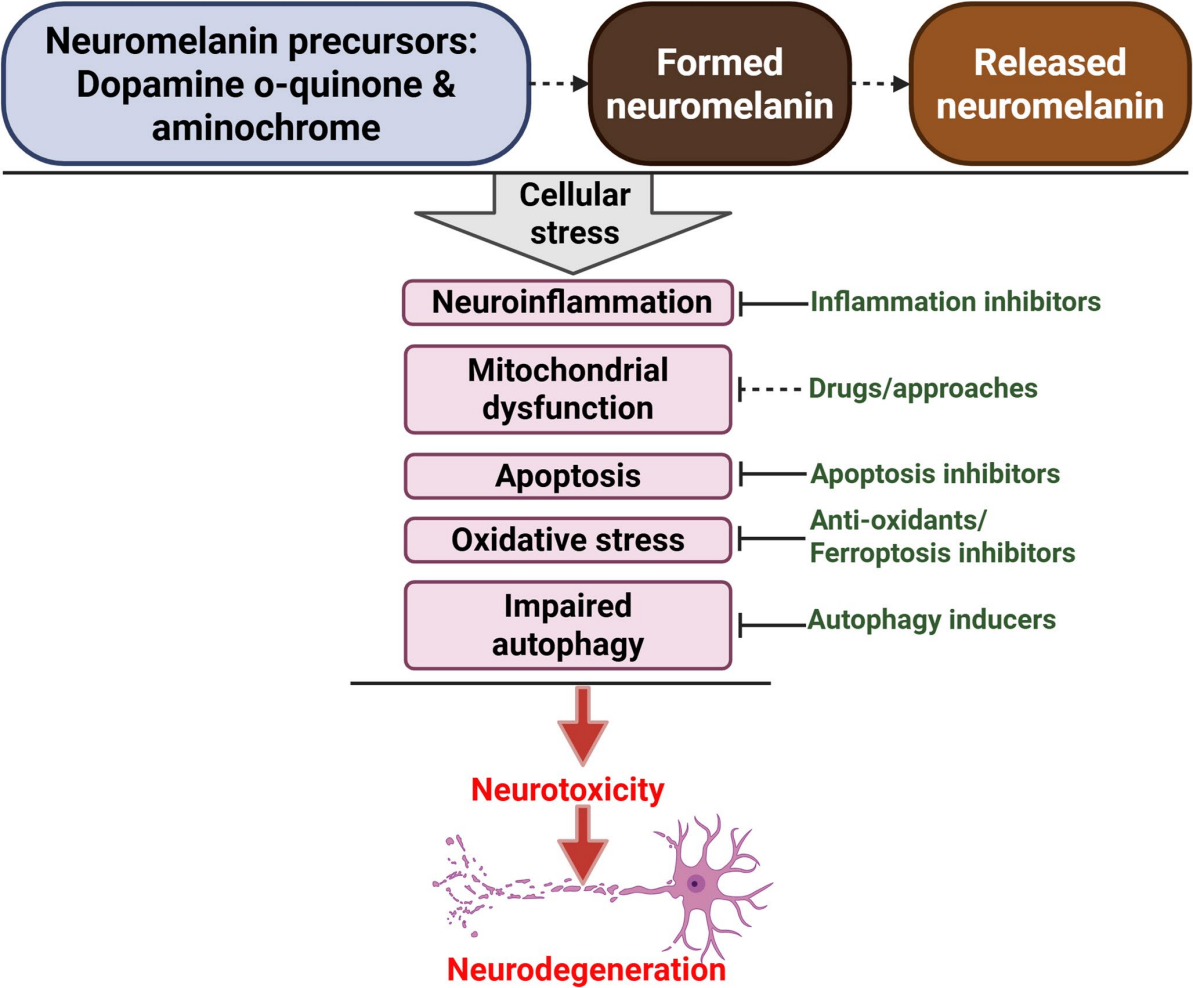
A summary of strategies aimed at combating cellular stress and neurotoxicity induced by neuromelanin. Neuromelanin and its precursors may trigger cellular stress. This can lead to dopaminergic neurotoxicity and neurodegeneration in PD. Several potential strategies could prevent this cellular stress, thereby protecting against neurodegeneration

Neuromelanin precursors, the synthesis pathway, and exogenous neuromelanin significantly contribute to dopaminergic neurotoxicity by inducing cellular stress (Fig. 4). This stress includes various processes such as neuroinflammation, apoptosis, oxidative stress, mitochondrial dysfunction, and impaired autophagy. While the neuromelanin precursors dopamine o-quinone and aminochrome are known to cause cellular stress and neurotoxicity, the effects of other precursors, such as DHI and DHICA, on cellular stress and neurotoxicity remain unknown.

While neuromelanin has been shown to interact with and influence α-synuclein-associated toxicity, it remains to be investigated whether it also interacts with other PD genetic risk factors, such as leucine-rich repeat kinase 2 (LRRK2), PTEN-induced kinase 1 (PINK1), and Parkin. Given the role of neuromelanin in causing impaired autophagy and mitochondrial dysfunction, it is necessary to test whether interactions between neuromelanin and these genes exacerbate impaired mitophagy-driven PD pathology.

The interaction of neuromelanin with harmful molecules such as α-synuclein, MPP+, harmane, PhIP, paraquat, iron, and aluminum can worsen neurotoxicity. However, the mechanisms underlying these interactions require further investigation. It is still uncertain whether the interactions of neuromelanin with other toxic substances, such as L-BMAA, contribute to or aggravate cellular stress and dopaminergic neurotoxicity.

Given the role of neuromelanin in neurodegeneration, we have outlined several protective strategies (Fig. 4) that could be explored as potential neuroprotective therapies for neuromelanin-associated diseases, such as PD. Since mitochondrial dysfunction is often linked to neuromelanin and its precursors, further research into pharmacological agents that target mitochondria would be beneficial. These drugs may help reduce the degree of cellular damage caused by the interactions between neuromelanin and mitochondrial impairments, thereby opening up new possibilities for therapeutic interventions in related neurological conditions.

While a tyrosinase-overexpressing model could be used to screen neuroprotective drugs, it has limitations due to the low expression of tyrosinase in the brain. Therefore, caution is needed when interpreting the neuroprotective effects of tyrosinase inhibitors, as these compounds may also exhibit antioxidant and anti-inflammatory properties. It is essential to test drugs for their neuroprotective effects against neuromelanin-associated damage rather than focusing solely on targeting tyrosinase in tyrosinase-overexpressing models.

Up to 60% of dopaminergic neurons may be irreversibly damaged by the time PD is diagnosed, highlighting the urgent need for a proactive approach to treatment. This underscores the critical importance of neuroprotective therapies, which would yield the greatest benefits if administered during the early stages of the disease. Consequently, research aimed at the early detection of PD has become paramount, with an emphasis on developing reliable biomarkers. Such advancements could significantly improve patient outcomes and pave the way for timely interventions, ultimately enhancing the quality of life for at-risk people.

## Data Availability

All raw data will be made available upon reasonable request.

## References

[1] Fedorow H, Tribl F, Halliday G, Gerlach M, Riederer P, Double KL (2005) Neuromelanin in human dopamine neurons: comparison with peripheral melanins and relevance to Parkinson’s disease. Prog Neurobiol 75:109–124 15784302 10.1016/j.pneurobio.2005.02.001

[2] Zucca FA, Basso E, Cupaioli FA et al (2014) Neuromelanin of the human substantia nigra: an update. Neurotox Res 25:13–23 24155156 10.1007/s12640-013-9435-y

[3] Xu Y, Stokes AH, Freeman WM, Kumer SC, Vogt BA, Vrana KE (1997) Tyrosinase mRNA is expressed in human substantia nigra. Brain Res Mol Brain Res 45:159–162 9105685 10.1016/s0169-328x(96)00308-7

[4] Tief K, Schmidt A, Beermann F (1998) New evidence for presence of tyrosinase in substantia nigra, forebrain and midbrain. Brain Res Mol Brain Res 53:307–310 9473705 10.1016/s0169-328x(97)00301-x

[5] Zecca L, Zucca FA, Wilms H, Sulzer D (2003) Neuromelanin of the substantia nigra: a neuronal black hole with protective and toxic characteristics. Trends Neurosci 26:578–580 14585596 10.1016/j.tins.2003.08.009

[6] Matsunaga J, Sinha D, Pannell L et al (1999) Enzyme activity of macrophage migration inhibitory factor toward oxidized catecholamines. J Biol Chem 274:3268–3271 9920865 10.1074/jbc.274.6.3268

[7] Hastings TG (1995) Enzymatic oxidation of dopamine: the role of prostaglandin H synthase. J Neurochem 64:919–924 7830086 10.1046/j.1471-4159.1995.64020919.x

[8] Fornstedt B, Rosengren E, Carlsson A (1986) Occurrence and distribution of 5-S-cysteinyl derivatives of dopamine, dopa and dopac in the brains of eight mammalian species. Neuropharmacology 25:451–454 3086766 10.1016/0028-3908(86)90242-x

[9] Liu L, Wang T, Zhou H et al (2024) Protective and damaging mechanisms of neuromelanin-like nanoparticles and Iron in Parkinson’s disease. Adv Healthc Mater 13:e2402718 39358952 10.1002/adhm.202402718

[10] Bellinger FP, Bellinger MT, Seale LA et al (2011) Glutathione peroxidase 4 is associated with neuromelanin in Substantia nigra and dystrophic axons in putamen of Parkinson’s brain. Mol Neurodegener 6:8 21255396 10.1186/1750-1326-6-8 PMC3037910

[11] NINDS. Parkinson's Disease

[12] Payami H, Cohen G, Murchison CF, Sampson TR, Standaert DG, Wallen ZD (2023) Population fraction of Parkinson’s disease attributable to preventable risk factors. NPJ Parkinsons Dis 9:159 38052871 10.1038/s41531-023-00603-z PMC10698155

[13] Cannon JR, Greenamyre JT (2013) Gene-environment interactions in Parkinson’s disease: specific evidence in humans and mammalian models. Neurobiol Dis 57:38–46 22776331 10.1016/j.nbd.2012.06.025 PMC3815566

[14] Olanow CW (2007) The pathogenesis of cell death in Parkinson’s disease–2007. Mov Disord 22(Suppl 17):S335-342 18175394 10.1002/mds.21675

[15] Hou X, Watzlawik JO, Fiesel FC, Springer W (2020) Autophagy in Parkinson’s disease. J Mol Biol 432:2651–2672 32061929 10.1016/j.jmb.2020.01.037 PMC7211126

[16] Hirsch E, Graybiel AM, Agid YA (1988) Melanized dopaminergic neurons are differentially susceptible to degeneration in Parkinson’s disease. Nature 334:345–348 2899295 10.1038/334345a0

[17] Hirsch EC, Graybiel AM, Agid Y (1989) Selective vulnerability of pigmented dopaminergic neurons in Parkinson’s disease. Acta Neurol Scand Suppl 126:19–22 2575832 10.1111/j.1600-0404.1989.tb01778.x

[18] Carballo-Carbajal I, Laguna A, Romero-Gimenez J et al (2019) Brain tyrosinase overexpression implicates age-dependent neuromelanin production in Parkinson’s disease pathogenesis. Nat Commun 10:973 30846695 10.1038/s41467-019-08858-y PMC6405777

[19] Shamoto-Nagai M, Maruyama W, Akao Y et al (2004) Neuromelanin inhibits enzymatic activity of 26S proteasome in human dopaminergic SH-SY5Y cells. J Neural Transm (Vienna) 111:1253–1265 15480837 10.1007/s00702-004-0211-2

[20] Chocarro J, Rico AJ, Ariznabarreta G et al (2023) Neuromelanin accumulation drives endogenous synucleinopathy in non-human primates. Brain 146:5000–5014 37769648 10.1093/brain/awad331 PMC10689915

[21] Viceconte N, Burguillos MA, Herrera AJ, De Pablos RM, Joseph B, Venero JL (2015) Neuromelanin activates proinflammatory microglia through a caspase-8-dependent mechanism. J Neuroinflammation 12:5 25586882 10.1186/s12974-014-0228-x PMC4302615

[22] Zecca L, Wilms H, Geick S et al (2008) Human neuromelanin induces neuroinflammation and neurodegeneration in the rat substantia nigra: implications for Parkinson’s disease. Acta Neuropathol 116:47–55 18343932 10.1007/s00401-008-0361-7

[23] Shamoto-Nagai M, Maruyama W, Yi H et al (2006) Neuromelanin induces oxidative stress in mitochondria through release of iron: mechanism behind the inhibition of 26S proteasome. J Neural Transm (Vienna) 113:633–644 16362626 10.1007/s00702-005-0410-5

[24] Naoi M, Maruyama W, Yi H et al (2008) Neuromelanin selectively induces apoptosis in dopaminergic SH-SY5Y cells by deglutathionylation in mitochondria: involvement of the protein and melanin component. J Neurochem 105:2489–2500 18399961 10.1111/j.1471-4159.2008.05329.x

[25] D’Amato RJ, Lipman ZP, Snyder SH (1986) Selectivity of the parkinsonian neurotoxin MPTP: toxic metabolite MPP+ binds to neuromelanin. Science 231:987–989 3080808 10.1126/science.3080808

[26] D’Amato RJ, Alexander GM, Schwartzman RJ, Kitt CA, Price DL, Snyder SH (1987) Evidence for neuromelanin involvement in MPTP-induced neurotoxicity. Nature 327:324–326 2884568 10.1038/327324a0

[27] Lawana V, Um SY, Rochet JC, Turesky RJ, Shannahan JH, Cannon JR (2020) Neuromelanin modulates heterocyclic aromatic amine-induced dopaminergic neurotoxicity. Toxicol Sci 173:171–188 31562763 10.1093/toxsci/kfz210 PMC6944224

[28] Channer B, Matt SM, Nickoloff-Bybel EA et al (2023) Dopamine, immunity, and disease. Pharmacol Rev 75:62–158 36757901 10.1124/pharmrev.122.000618 PMC9832385

[29] Berman SB, Hastings TG (1999) Dopamine oxidation alters mitochondrial respiration and induces permeability transition in brain mitochondria: implications for Parkinson’s disease. J Neurochem 73:1127–1137 10461904 10.1046/j.1471-4159.1999.0731127.x

[30] Klegeris A, Korkina LG, Greenfield SA (1995) Autoxidation of dopamine: a comparison of luminescent and spectrophotometric detection in basic solutions. Free Radic Biol Med 18:215–222 7744304 10.1016/0891-5849(94)00141-6

[31] Burbulla LF, Song P, Mazzulli JR et al (2017) Dopamine oxidation mediates mitochondrial and lysosomal dysfunction in Parkinson’s disease. Science 357:1255–1261 28882997 10.1126/science.aam9080 PMC6021018

[32] Masserano JM, Baker I, Venable D et al (2000) Dopamine induces cell death, lipid peroxidation and DNA base damage in a catecholaminergic cell line derived from the central nervous system. Neurotox Res 1:171–179 12835100 10.1007/BF03033288

[33] Segura-Aguilar J, Munoz P, Inzunza J, Varshney M, Nalvarte I, Mannervik B (2022) Neuroprotection against aminochrome neurotoxicity: glutathione transferase M2–2 and DT-diaphorase. Antioxidants (Basel). 10.3390/antiox11020296 35204179 10.3390/antiox11020296 PMC8868244

[34] Sulzer D, Cassidy C, Horga G et al (2018) Neuromelanin detection by magnetic resonance imaging (MRI) and its promise as a biomarker for Parkinson’s disease. NPJ Parkinsons Dis 4:11 29644335 10.1038/s41531-018-0047-3 PMC5893576

[35] Munoz P, Huenchuguala S, Paris I, Segura-Aguilar J (2012) Dopamine oxidation and autophagy. Parkinsons Dis. 10.1155/2012/920953 22966478 10.1155/2012/920953 PMC3433151

[36] Paris I, Cardenas S, Lozano J et al (2007) Aminochrome as a preclinical experimental model to study degeneration of dopaminergic neurons in Parkinson’s disease. Neurotox Res 12:125–134 17967736 10.1007/BF03033921

[37] Segura-Aguilar J, Lind C (1989) On the mechanism of the Mn3(+)-induced neurotoxicity of dopamine:prevention of quinone-derived oxygen toxicity by DT diaphorase and superoxide dismutase. Chem Biol Interact 72:309–324 2557982 10.1016/0009-2797(89)90006-9

[38] Arriagada C, Paris I, de las Matas MJ et al (2004) On the neurotoxicity mechanism of leukoaminochrome o-semiquinone radical derived from dopamine oxidation: mitochondria damage, necrosis, and hydroxyl radical formation. Neurobiol Dis 16:468–477 15193303 10.1016/j.nbd.2004.03.014

[39] Lozano J, Munoz P, Nore BF, Ledoux S, Segura-Aguilar J (2010) Stable expression of short interfering RNA for DT-diaphorase induces neurotoxicity. Chem Res Toxicol 23:1492–1496 20849151 10.1021/tx100182a

[40] de Araujo FM, Ferreira RS, Souza CS et al (2018) Aminochrome decreases NGF, GDNF and induces neuroinflammation in organotypic midbrain slice cultures. Neurotoxicology 66:98–106 29588162 10.1016/j.neuro.2018.03.009

[41] De Araujo FM, Frota AF, de Jesus LB et al (2023) Aminochrome induces neuroinflammation and dopaminergic neuronal loss: a new preclinical model to find anti-inflammatory and neuroprotective drugs for Parkinson’s disease. Cell Mol Neurobiol 43:265–281 34988761 10.1007/s10571-021-01173-5 PMC11415180

[42] Norris EH, Giasson BI, Hodara R et al (2005) Reversible inhibition of alpha-synuclein fibrillization by dopaminochrome-mediated conformational alterations. J Biol Chem 280:21212–21219 15817478 10.1074/jbc.M412621200

[43] Munoz P, Cardenas S, Huenchuguala S et al (2015) DT-diaphorase prevents aminochrome-induced alpha-synuclein oligomer formation and neurotoxicity. Toxicol Sci 145:37–47 25634539 10.1093/toxsci/kfv016 PMC4833033

[44] Huenchuguala S, Munoz P, Zavala P et al (2014) Glutathione transferase mu 2 protects glioblastoma cells against aminochrome toxicity by preventing autophagy and lysosome dysfunction. Autophagy 10:618–630 24434817 10.4161/auto.27720 PMC4091149

[45] Aguirre P, Urrutia P, Tapia V et al (2012) The dopamine metabolite aminochrome inhibits mitochondrial complex I and modifies the expression of iron transporters DMT1 and FPN1. Biometals 25:795–803 22302610 10.1007/s10534-012-9525-y

[46] Herrera A, Munoz P, Paris I et al (2016) Aminochrome induces dopaminergic neuronal dysfunction: a new animal model for Parkinson’s disease. Cell Mol Life Sci 73:3583–3597 27001668 10.1007/s00018-016-2182-5 PMC11108377

[47] Paris I, Munoz P, Huenchuguala S et al (2011) Autophagy protects against aminochrome-induced cell death in substantia nigra-derived cell line. Toxicol Sci 121:376–388 21427056 10.1093/toxsci/kfr060 PMC3145404

[48] Hasegawa T (2010) Tyrosinase-expressing neuronal cell line as in vitro model of Parkinson’s disease. Int J Mol Sci 11:1082–1089 20480001 10.3390/ijms11031082 PMC2869230

[49] Iannitelli AF, Hassenein L, Mulvey B, et al. (2024) Tyrosinase-induced neuromelanin accumulation triggers rapid dysregulation and degeneration of the mouse locus coeruleus. bioRxiv

[50] Gonzalez-Sepulveda M, Compte J, Cuadros T et al (2023) In vivo reduction of age-dependent neuromelanin accumulation mitigates features of Parkinson’s disease. Brain 146:1040–1052 36717986 10.1093/brain/awac445 PMC9976971

[51] Laguna A, Penuelas N, Gonzalez-Sepulveda M et al (2024) Modelling human neuronal catecholaminergic pigmentation in rodents recapitulates age-related neurodegenerative deficits. Nat Commun 15:8819 39394193 10.1038/s41467-024-53168-7 PMC11470033

[52] Miranda M, Botti D, Bonfigli A, Ventura T, Arcadi A (1984) Tyrosinase-like activity in normal human substantia nigra. Gen Pharmacol 15:541–544 6441736 10.1016/0306-3623(84)90212-x

[53] Greggio E, Bergantino E, Carter D et al (2005) Tyrosinase exacerbates dopamine toxicity but is not genetically associated with Parkinson’s disease. J Neurochem 93:246–256 15773923 10.1111/j.1471-4159.2005.03019.x

[54] Li J, Yang J, Zhao P et al (2012) Neuromelanin enhances the toxicity of alpha-synuclein in SK-N-SH cells. J Neural Transm (Vienna) 119:685–691 22200858 10.1007/s00702-011-0753-z

[55] Tejchman-Skrzyszewska A, Strzelec M, Kot M, et al. (2023) Synthetic neuromelanin as a trigger of inflammation in the brain– new mouse model of Parkinson’s disease. bioRxiv:2023.2004.2028.538536.

[56] Wilms H, Rosenstiel P, Sievers J, Deuschl G, Zecca L, Lucius R (2003) Activation of microglia by human neuromelanin is NF-kappaB dependent and involves p38 mitogen-activated protein kinase: implications for Parkinson’s disease. FASEB J 17:500–502 12631585 10.1096/fj.02-0314fje

[57] Zhang W, Zecca L, Wilson B et al (2013) Human neuromelanin: an endogenous microglial activator for dopaminergic neuron death. Front Biosci (Elite Ed) 5:1–11 23276965 10.2741/e591 PMC3626451

[58] Oberlander U, Pletinckx K, Dohler A et al (2011) Neuromelanin is an immune stimulator for dendritic cells in vitro. BMC Neurosci 12:116 22085464 10.1186/1471-2202-12-116 PMC3225309

[59] Dehay B, Bové J, Rodríguez-Muela N et al (2010) Pathogenic lysosomal depletion in Parkinson’s disease. J Neurosci 30:12535–12544 20844148 10.1523/JNEUROSCI.1920-10.2010 PMC6633458

[60] McNaught KS, Belizaire R, Isacson O, Jenner P, Olanow CW (2003) Altered proteasomal function in sporadic Parkinson’s disease. Exp Neurol 179:38–46 12504866 10.1006/exnr.2002.8050

[61] Vila M, Laguna A, Carballo-Carbajal I (2019) Intracellular crowding by age-dependent neuromelanin accumulation disrupts neuronal proteostasis and triggers Parkinson disease pathology. Autophagy 15:2028–2030 31480882 10.1080/15548627.2019.1659621 PMC6844506

[62] Xuan Q, Xu SL, Lu DH et al (2011) Increased expression of alpha-synuclein in aged human brain associated with neuromelanin accumulation. J Neural Transm (Vienna) 118:1575–1583 21461961 10.1007/s00702-011-0636-3

[63] Grundemann J, Schlaudraff F, Liss B (2011) UV-laser microdissection and mRNA expression analysis of individual neurons from postmortem Parkinson’s disease brains. Methods Mol Biol 755:363–374 21761319 10.1007/978-1-61779-163-5_30

[64] Halliday GM, Ophof A, Broe M et al (2005) Alpha-synuclein redistributes to neuromelanin lipid in the substantia nigra early in Parkinson’s disease. Brain 128:2654–2664 16000336 10.1093/brain/awh584

[65] Cole NB, Murphy DD, Grider T, Rueter S, Brasaemle D, Nussbaum RL (2002) Lipid droplet binding and oligomerization properties of the Parkinson’s disease protein alpha-synuclein. J Biol Chem 277:6344–6352 11744721 10.1074/jbc.M108414200

[66] Pan T, Zhu J, Hwu WJ, Jankovic J (2012) The role of alpha-synuclein in melanin synthesis in melanoma and dopaminergic neuronal cells. PLoS ONE 7:e45183 23028833 10.1371/journal.pone.0045183 PMC3446957

[67] Garcia Moreno SI, Limani F, Ludwig I et al (2024) Viral overexpression of human alpha-synuclein in mouse substantia nigra dopamine neurons results in hyperdopaminergia but no neurodegeneration. Exp Neurol 382:114959 39288832 10.1016/j.expneurol.2024.114959

[68] Cascella R, Chen SW, Bigi A et al (2021) The release of toxic oligomers from alpha-synuclein fibrils induces dysfunction in neuronal cells. Nat Commun 12:1814 33753734 10.1038/s41467-021-21937-3 PMC7985515

[69] Stykel MG, Siripala SV, Soubeyrand E et al (2025) G6PD deficiency triggers dopamine loss and the initiation of Parkinson’s disease pathogenesis. Cell Rep 44:115178 39772392 10.1016/j.celrep.2024.115178

[70] Jakaria M, Belaidi AA, Bush AI, Ayton S (2023) Vitamin A metabolites inhibit ferroptosis. Biomed Pharmacother 164:114930 37236031 10.1016/j.biopha.2023.114930

[71] Jakaria M, Belaidi AA, Southon A et al (2022) Receptor-independent anti-ferroptotic activity of TrkB modulators. Int J Mol Sci 23:16205 36555849 10.3390/ijms232416205 PMC9784883

[72] Jakaria M, Belaidi AA, Bush AI, Ayton S (2021) Ferroptosis as a mechanism of neurodegeneration in Alzheimer’s disease. J Neurochem 159:804–825 34553778 10.1111/jnc.15519

[73] Mahoney-Sanchez L, Bouchaoui H, Boussaad I et al (2022) Alpha synuclein determines ferroptosis sensitivity in dopaminergic neurons via modulation of ether-phospholipid membrane composition. Cell Rep 40:111231 36001957 10.1016/j.celrep.2022.111231

[74] Angelova PR, Choi ML, Berezhnov AV et al (2020) Alpha synuclein aggregation drives ferroptosis: an interplay of iron, calcium and lipid peroxidation. Cell Death Differ 27:2781–2796 32341450 10.1038/s41418-020-0542-z PMC7492459

[75] Yildirim-Balatan C, Fenyi A, Besnault P et al (2024) Parkinson’s disease-derived alpha-synuclein assemblies combined with chronic-type inflammatory cues promote a neurotoxic microglial phenotype. J Neuroinflammation 21:54 38383421 10.1186/s12974-024-03043-5 PMC10882738

[76] Davis GC, Williams AC, Markey SP et al (1979) Chronic parkinsonism secondary to intravenous injection of meperidine analogues. Psychiatry Res 1:249–254 298352 10.1016/0165-1781(79)90006-4

[77] Langston JW, Ballard P, Tetrud JW, Irwin I (1983) Chronic parkinsonism in humans due to a product of meperidine-analog synthesis. Science 219:979–980 6823561 10.1126/science.6823561

[78] Herrero MT, Hirsch EC, Kastner A et al (1993) Does neuromelanin contribute to the vulnerability of catecholaminergic neurons in monkeys intoxicated with MPTP? Neuroscience 56:499–511 8247275 10.1016/0306-4522(93)90349-k

[79] Lindquist NG, Larsson BS, Lyden-Sokolowski A (1988) Autoradiography of [14C]paraquat or [14C]diquat in frogs and mice: accumulation in neuromelanin. Neurosci Lett 93:1–6 3264893 10.1016/0304-3940(88)90002-x

[80] D’Amato RJ, Benham DF, Snyder SH (1987) Characterization of the binding of N-methyl-4-phenylpyridine, the toxic metabolite of the parkinsonian neurotoxin N-methyl-4-phenyl-1,2,3,6-tetrahydropyridine, to neuromelanin. J Neurochem 48:653–658 3491879 10.1111/j.1471-4159.1987.tb04142.x

[81] John K (2017) Heterocyclic Amines. In: Schwab M (ed) Encyclopedia of Cancer. Springer Berlin Heidelberg, Berlin, Heidelberg, pp 2066–2072

[82] Syeda T, Cannon JR (2022) Potential role of heterocyclic aromatic amines in neurodegeneration. Chem Res Toxicol 35:59–72 34990108 10.1021/acs.chemrestox.1c00274 PMC10443083

[83] Knize MG, Salmon CP, Pais P, Felton JS (1999) Food heating and the formation of heterocyclic aromatic amine and polycyclic aromatic hydrocarbon mutagens/carcinogens. In: Jackson LS, Knize MG, Morgan JN (eds) Impact of processing on food safety. Springer, US, Boston, MA, pp 179–193 10.1007/978-1-4615-4853-9_12 10335376

[84] Lawana V, Um SY, Foguth RM, Cannon JR (2020) Neuromelanin formation exacerbates HAA-induced mitochondrial toxicity and mitophagy impairments. Neurotoxicology 81:147–160 33058929 10.1016/j.neuro.2020.10.005 PMC7708437

[85] Lobner D (2009) Mechanisms of beta-N-methylamino-L-alanine induced neurotoxicity. Amyotroph Lateral Scler 10(Suppl 2):56–60 19929733 10.3109/17482960903269062

[86] Karlsson O, Berg C, Brittebo EB, Lindquist NG (2009) Retention of the cyanobacterial neurotoxin beta-N-methylamino-l-alanine in melanin and neuromelanin-containing cells–a possible link between Parkinson-dementia complex and pigmentary retinopathy. Pigment Cell Melanoma Res 22:120–130 19154235 10.1111/j.1755-148X.2008.00508.x

[87] Zecca L, Bellei C, Costi P et al (2008) New melanic pigments in the human brain that accumulate in aging and block environmental toxic metals. Proc Natl Acad Sci U S A 105:17567–17572 18988735 10.1073/pnas.0808768105 PMC2582310

[88] Capucciati A, Zucca FA, Monzani E, Zecca L, Casella L, Hofer T (2021) Interaction of neuromelanin with xenobiotics and consequences for neurodegeneration; promising Experimental Models. Antioxidants (Basel) 10:824 34064062 10.3390/antiox10060824 PMC8224073

[89] Faucheux BA, Martin ME, Beaumont C, Hauw JJ, Agid Y, Hirsch EC (2003) Neuromelanin associated redox-active iron is increased in the substantia nigra of patients with Parkinson’s disease. J Neurochem 86:1142–1148 12911622 10.1046/j.1471-4159.2003.01923.x

[90] Jellinger K, Kienzl E, Rumpelmair G et al (1992) Iron-melanin complex in substantia nigra of parkinsonian brains: an x-ray microanalysis. J Neurochem 59:1168–1171 1494904 10.1111/j.1471-4159.1992.tb08362.x

[91] Meglio L, Oteiza PI (1999) Aluminum enhances melanin-induced lipid peroxidation. Neurochem Res 24:1001–1008 10478939 10.1023/a:1021000709082

[92] Sulzer D, Bogulavsky J, Larsen KE et al (2000) Neuromelanin biosynthesis is driven by excess cytosolic catecholamines not accumulated by synaptic vesicles. Proc Natl Acad Sci U S A 97:11869–11874 11050221 10.1073/pnas.97.22.11869 PMC17261

[93] Devos D, Moreau C, Devedjian JC et al (2014) Targeting chelatable iron as a therapeutic modality in Parkinson’s disease. Antioxid Redox Signal 21:195–210 24251381 10.1089/ars.2013.5593 PMC4060813

[94] Devos D, Labreuche J, Rascol O et al (2022) Trial of deferiprone in Parkinson’s disease. N Engl J Med 387:2045–2055 36449420 10.1056/NEJMoa2209254

[95] Offen D, Ziv I, Sternin H, Melamed E, Hochman A (1996) Prevention of dopamine-induced cell death by thiol antioxidants: possible implications for treatment of Parkinson’s disease. Exp Neurol 141:32–39 8797665 10.1006/exnr.1996.0136

[96] Khan A, Park TJ, Ikram M et al (2021) Antioxidative and anti-inflammatory effects of kojic acid in Abeta-induced mouse model of Alzheimer’s disease. Mol Neurobiol 58:5127–5140 34255249 10.1007/s12035-021-02460-4

[97] Ali W, Choe K, Park JS et al (2024) Kojic acid reverses LPS-induced neuroinflammation and cognitive impairment by regulating the TLR4/NF-kappaB signaling pathway. Front Pharmacol 15:1443552 39185307 10.3389/fphar.2024.1443552 PMC11341365

[98] Li Q, Mo J, Xiong B et al (2022) Discovery of resorcinol-based polycyclic structures as tyrosinase inhibitors for treatment of Parkinson’s disease. ACS Chem Neurosci 13:81–96 34882402 10.1021/acschemneuro.1c00560

[99] Qi S, Guo L, Liang J et al (2024) A new strategy for the treatment of Parkinson’s disease: discovery and bio-evaluation of the first central-targeting tyrosinase inhibitor. Bioorg Chem 150:107612 38986418 10.1016/j.bioorg.2024.107612

[100] Zuo L, Dai C, Yi L, Dong Z (2021) 7,8-dihydroxyflavone ameliorates motor deficits via regulating autophagy in MPTP-induced mouse model of Parkinson’s disease. Cell Death Discov 7:254 34545064 10.1038/s41420-021-00643-5 PMC8452727

[101] Cai W, Wang J, Hu M et al (2019) All trans-retinoic acid protects against acute ischemic stroke by modulating neutrophil functions through STAT1 signaling. J Neuroinflammation 16:175 31472680 10.1186/s12974-019-1557-6 PMC6717357

[102] Hummel R, Ulbrich S, Appel D et al (2020) Administration of all-trans retinoic acid after experimental traumatic brain injury is brain protective. Br J Pharmacol 177:5208–5223 32964418 10.1111/bph.15259 PMC7588818

[103] Hill RL, Singh IN, Wang JA, Hall ED (2019) Effects of phenelzine administration on mitochondrial function, calcium handling, and cytoskeletal degradation after experimental traumatic brain injury. J Neurotrauma 36:1231–1251 30358485 10.1089/neu.2018.5946 PMC6479250

[104] Garcia-Gomara M, Juan-Palencia A, Alfaro M, Cuadrado-Tejedor M, Garcia-Osta A (2024) Neuroprotective effects of dexamethasone in a neuromelanin-driven Parkinson’s disease model. J Neuroimmune Pharmacol 20:2 39672994 10.1007/s11481-024-10164-4 PMC11645310

[105] Malagelada C, Jin ZH, Jackson-Lewis V, Przedborski S, Greene LA (2010) Rapamycin protects against neuron death in in vitro and in vivo models of Parkinson’s disease. J Neurosci 30:1166–1175 20089925 10.1523/JNEUROSCI.3944-09.2010 PMC2880868

[106] Pupyshev AB, Tikhonova MA, Akopyan AA, Tenditnik MV, Dubrovina NI, Korolenko TA (2019) Therapeutic activation of autophagy by combined treatment with rapamycin and trehalose in a mouse MPTP-induced model of Parkinson’s disease. Pharmacol Biochem Behav 177:1–11 30582934 10.1016/j.pbb.2018.12.005

[107] Hirata Y, Yamamoto H, Atta MS, Mahmoud S, Oh-hashi K, Kiuchi K (2011) Chloroquine inhibits glutamate-induced death of a neuronal cell line by reducing reactive oxygen species through sigma-1 receptor. J Neurochem 119:839–847 21883227 10.1111/j.1471-4159.2011.07464.x

[108] Cui CM, Gao JL, Cui Y et al (2015) Chloroquine exerts neuroprotection following traumatic brain injury via suppression of inflammation and neuronal autophagic death. Mol Med Rep 12:2323–2328 25872478 10.3892/mmr.2015.3611

[109] Kartik S, Pal R, Chaudhary MJ et al (2023) Neuroprotective role of chloroquine via modulation of autophagy and neuroinflammation in MPTP-induced Parkinson’s disease. Inflammopharmacology 31:927–941 36715843 10.1007/s10787-023-01141-z

[110] Smedlund KB, Sanchez ER, Hinds TD Jr. (2021) FKBP51 and the molecular chaperoning of metabolism. Trends Endocrinol Metab 32:862–874 34481731 10.1016/j.tem.2021.08.003 PMC8516732

[111] Garcia-Gomara M, Legarra-Marcos N, Serena M et al (2025) FKBP51 inhibition ameliorates neurodegeneration and motor dysfunction in the neuromelanin-SNCA mouse model of Parkinson’s disease. Mol Ther 33:895–916 39905728 10.1016/j.ymthe.2025.01.049 PMC11897814

[112] Herrera-Soto A, Diaz-Veliz G, Mora S et al (2017) On the role of DT-diaphorase inhibition in aminochrome-induced neurotoxicity in vivo. Neurotox Res 32:134–140 28285345 10.1007/s12640-017-9719-8

[113] Huenchuguala S, Munoz P, Graumann R, Paris I, Segura-Aguilar J (2016) DT-diaphorase protects astrocytes from aminochrome-induced toxicity. Neurotoxicology 55:10–12 27168424 10.1016/j.neuro.2016.04.014

[114] Bernstein AI, Stout KA, Miller GW (2014) The vesicular monoamine transporter 2: an underexplored pharmacological target. Neurochem Int 73:89–97 24398404 10.1016/j.neuint.2013.12.003 PMC5028832

[115] Pifl C, Rajput A, Reither H et al (2014) Is Parkinson’s disease a vesicular dopamine storage disorder? Evidence from a study in isolated synaptic vesicles of human and nonhuman primate striatum. J Neurosci 34:8210–8218 24920625 10.1523/JNEUROSCI.5456-13.2014 PMC6608236

[116] Liang CL, Nelson O, Yazdani U, Pasbakhsh P, German DC (2004) Inverse relationship between the contents of neuromelanin pigment and the vesicular monoamine transporter-2: human midbrain dopamine neurons. J Comp Neurol 473:97–106 15067721 10.1002/cne.20098

